# Plant species richness and phylogenetic diversity can favor the recovery of dung beetle communities in ecological restoration plots

**DOI:** 10.1007/s00442-025-05666-8

**Published:** 2025-02-01

**Authors:** Lina Adonay Urrea-Galeano, Rocío Santos-Gally, José D. Rivera-Duarte, Alfonso Díaz Rojas, Karina Boege

**Affiliations:** 1 Instituto de Ecología, Universidad Nacional Autónoma de México, Ciudad de Mexico, Mexico; 2 CONAHCYT-Instituto de Ecología, Departamento de Ecología Evolutiva, Universidad Nacional Autónoma de México, Ciudad de Mexico, Mexico; 3 Instituto de Ecología, A.C., Xalapa, Veracruz Mexico; 4 Laboratorio de Hidrobiología, Departamento de Ecología y Recursos Naturales, Escuela de Biología, Facultad de Ciencias, Universidad Nacional Autónoma de Honduras, Tegucigalpa, M.D.C. Francisco Morazán Honduras

**Keywords:** Diversity, Ecological restoration, Habitat heterogeneity, Microclimate, Niche opportunities, Scarabaeinae

## Abstract

Plant communities with higher species richness and phylogenetic diversity can increase the diversity of herbivores and their enemies through trophic interactions. However, whether these two features of plant communities have the same positive influence on other guilds through non-trophic mechanisms requires further exploration. Dung beetles represent an ideal system for testing such impacts, as they do not have a specialized trophic interaction with plants and are sensitive to changes in vegetation structure and the associated microclimate. We used a dataset of dung beetles collected from forest sites, restoration plots, and cattle pastures to (a) determine whether the richness and phylogenetic diversity of plants within restoration plots influence the total biomass and the taxonomic, functional, and phylogenetic diversity of dung beetles; and (b) determine if the establishment of restoration plots allows to recover the abundance and diversity of dung beetle communities, relative to what is found in livestock pastures. In the restoration plots, the abundance of Scarabaeinae beetles and the total biomass, functional originality, and phylogenetic diversity of dung beetles were positively related to the number of plant species, but only the abundance of Scarabaeinae and total biomass of all dung beetles were positively related to the plant phylogenetic diversity. Finally, the restoration plots allowed a threefold increase in the total biomass of dung beetles relative to the biomass found in pastures. We discuss how restoration plots with high plant species richness and phylogenetic diversity can favor the recovery of dung beetle communities by potentially creating more niche opportunities.

**Supplementary Information:**

The online version contains supplementary material available at 10.1007/s00442-025-05666-8.

## Introduction

Active restoration plots within productive livestock ranches can improve landscape connectivity (de la Peña-Domene et al. 2016), and closely resemble the plant structure and diversity of primary forests (Werden et al. 2020; Beltrán et al. 2022). This recovery of vegetation structure can speed up forest succession, thereby fostering the recuperation of native fauna involved in different biotic interactions and ecological processes (Catterall 2018).

Framed within the biodiversity-ecosystem functioning theory (Tilman et al. 2014), we can expect communities with a high number of plant species to have a greater diversity of other taxa (Zhang et al. 2017; Brunbjerg et al. 2018; Fornoff et al. 2019), given an increase in the number and diversity of food resources (Fornoff et al. 2019). Furthermore, plant phylogenetic diversity accounts for the evolutionary history among species in a community incorporating their functional differences (Srivastava et al. 2012). Hence, communities with high diversity, and consequently more complementary ecological niches (Webb et al. 2002; Srivastava et al. 2012), can increase the diversity and composition of associated guilds across higher trophic levels. This enhancement occurs because phylogenetically diverse plant communities provide a wider array of resource diversity and niche opportunities (Staab et al. 2021). Positive relationships of plant species richness and phylogenetic diversity have been mainly evaluated for herbivorous arthropods that directly rely on plants as their food source (Lind et al. 2015; Zhang et al. 2017; Staab et al. 2021), but also for their predators (Dinnage et al. 2012; Ugalde et al. 2024) and parasitoids (Salazar et al. 2016; Alavez et al. 2023), which are influenced by prey availability, the diversity of refuges, and plant cues to find their prey. However, a few studies have also suggested that increased plant species richness and phylogenetic diversity can enhance the diversity of animals that do not rely directly on plants as food resources, possibly due to an increase in environmental heterogeneity (Skarbek et al. 2020; Staab et al. 2021) and ecological niches, or due to indirect effects on other resources (Raine and Slade 2019), which together promote species coexistence (Stein et al. 2014).

Among the animals influenced by environmental heterogeneity are dung beetles (Scarabaeidae: Scarabaeinae & Aphodiinae; Pessôa et al. 2021). These insects process dung for feeding or laying eggs (Hanski and Cambefort 1991). Scarabaeinae dung beetles, primarily distributed in tropical regions, display intricate nesting behaviors closely linked to dung manipulation (Halffter and Matthews 1966). They are classified as tunnellers, rollers, and dwellers. Tunnellers place dung below the pat, while rollers transport it away, and dwellers live inside or in the immediate vicinity below the pat (Halffter and Edmonds 1982). In contrast, Aphodiinae dung beetles exhibit mostly dwelling behaviors and inhabit primarily cold-temperate areas (Cabrero-Sañudo et al. 2010), although they also occur in Neotropical regions (Cajaiba et al. 2018). Through dung manipulation, dung beetles play key ecological functions in cattle grazing systems, including dung removal, pasture cleaning, nutrient cycling, soil bioturbation, and parasite suppression (Arellano et al. 2023). Therefore, dung removal constitutes an ecosystem process of high economic value, particularly in cattle ranches (Lopez-Collado et al. 2017).

Dung beetles can be influenced by vegetation structure which, in turn, determines microclimatic conditions such as moisture and temperature (Hanski and Cambefort 1991; Audino et al. 2017). In addition, leaf litter depth and cover can affect beetle diversity, biomass distribution, and nesting strategies (Nichols et al. 2013; Viegas et al. 2014; da Silva and Hernández 2016). Variation in vegetation structure and leaf litter characteristics can produce significant heterogeneity in microenvironmental conditions (Stein et al. 2014), supporting the persistence of species with diverse habitat requirements (Hanski and Cambefort 1991; Audino et al. 2017). Furthermore, because the amount of litter is directly influenced by the species composition of plant communities, previous studies have reported that habitats with greater vegetation cover support higher diversity and biomass of dung beetles (e.g., Rivera et al. 2021; Carvalho et al. 2023; Ratoni et al. 2023).

Most studies on dung beetle communities in restoration areas have focused on comparing restoration strategies (Díaz-García et al. 2020, 2022; Gelviz-Gelvez et al. 2023), timing of forest succession (Audino et al. 2014, 2017), or on the effect surrounding active cattle pastures (González-Tokman et al. 2018). However, the influence that plant species richness can have on the recovery of dung beetles in restoration plots has been examined in only one study, which found that at low plant density, increased tree diversity enhanced the taxonomic diversity of dung beetles as well as their ecological functions (i.e., dung removal, seed dispersal; Menéndez et al. 2024). The influence of plant phylogenetic diversity on dung beetle communities, however, remains unexplored.

Plant species richness is an important factor influencing vegetation structure (i.e., canopy cover; Fornoff et al. 2021; Xu et al. 2022) and leaf litter production (Alves Silva et al. 2020). Given that dung beetle communities can be modulated by the patchy and temporal distribution of suitable microhabitats (Hanski and Koskela 1977) and availability of food resources (e.g., dung, rotten fruits, and/or fungi; Halffter and Halffter 2009), higher plant diversity is likely to promote increased niche partitioning, leading to greater dung beetle diversity (Rivera et al. 2021). Furthermore, because plant phylogenetic diversity can influence biomass production and litter decomposition (Cadotte et al. 2008; Xiao et al. 2020), plant communities with higher phylogenetic diversity may provide more niches for dung beetles than those with lower diversity.

In this study, we assessed whether restoration plots in active cattle pastures allowed dung beetle communities to recover their taxonomic, functional, and phylogenetic diversity, as well as their biomass, as a function of plant species and phylogenetic diversity. To understand how dung beetles respond to plant diversity in restoration plots, we examined three dimensions of their diversity: 1) taxonomic diversity, which includes species richness and abundance (Gotelli and Colwell 2001); 2) functional diversity, which accounts for the variety of functional traits within a community (Mouillot et al. 2013); and 3) phylogenetic diversity, representing the mean evolutionary distance among species in a community (Webb et al. 2002). Additionally, we measured dung beetle biomass, which relates to beetle activity (Ratoni et al. 2023) and can be considered as an indirect measure of their dung processing capacity, a factor relevant to cattle ranching. Under the assumption that plant species and phylogenetic diversity influence vegetation structure, microclimate, and the availability of niches in leaf litter, we predicted that (1) taxonomic and phylogenetic plant diversity would positively influence dung beetle taxonomic, functional, and phylogenetic diversity and their total biomass, and (2) the establishment of restoration plots would promote the recovery of the three dimensions of dung beetle diversity and total biomass relative to cattle pastures.

## Methods

### Study site

We conducted our study in the cattle ranch “Los Amigos” (18° 32′ 59.4″ N, 95° 00′ 09.9″ W), located in the buffer zone of the Biosphere Reserve of Los Tuxtlas, Veracruz, Mexico. The climate in this area is tropical and humid, with a mean annual temperature of 24.6 °C and a mean annual precipitation of 3840 mm (Gutiérrez-García and Ricker 2011). The reserve encompasses 125,406 ha of buffer zone, where agricultural fields and pastures for cattle are predominant and interspersed with small forest fragments (i.e., tropical rainforest; von Thaden et al. 2020). Cattle ranching is the main economic activity in the area, and hence, pastures occupy 49.70% of the reserve area (155,122.5 ha; von Thaden et al. 2020).

To assess the influence of plant species richness and phylogenetic diversity on the metrics of dung beetle communities (Prediction 1), we collected dung beetles in June and August 2022 in 22 restoration plots. These 15 m × 15 m plots were established between August 2018 and January 2019 within an active cattle pasture matrix (Santos-Gally and Boege 2022). Plots were separated by ≥ 50 m and protected with an electric fence to prevent cattle from grazing. Within each plot, we planted 196 seedlings of 43 native tree species in a 1 m × 1 m matrix, although some of them failed to germinate or survive, resulting in a range of 21–28 species per plot. Because increasing phylogenetic diversity reduces the likelihood of finding closely related species within the same clade, plant arrays for each plot were chosen from the regional species pool to produce contrasting plant communities: half of the plots had high phylogenetic diversity (with 27 species from 23 families) and the other half had low phylogenetic diversity (with 27 species from 10 families; for further details, see Alavez et al. 2023). Because after 3.5 years, more plant species had naturally colonized these experimental plots, in May 2022 we carried out a vegetation census to define the resulting new plant communities and their phylogenetic diversity. According to the census, plant communities within each plot represented a gradient of phylogenetic diversity, with a total of 239 plant species belonging to 169 genera and 60 families, including planted and colonizing species. The mean number of plant species per plot was 58 (min = 39 spp., max = 87 spp.).

To assess the phylogenetic diversity of plant communities within the restoration plots, we used the species-level phylogenetic tree published by Smith and Brown (2018) and enhanced by Jin and Qian (2019), which includes 74,531 species of vascular plants. We employed V Phylomaker (Jin and Qian 2019) to generate the regional species phylogeny (290 species) from the phylogenetic tree mentioned above (GBOTB.extended.tre). We assessed the phylogenetic structure of each plant community using a standardized index (SES.MPD; see Null models section) of the mean pairwise distance (MPD), which estimates the pairwise phylogenetic distance between species in a community and it is equivalent to the net relatedness index (Webb et al. 2002). After calculating SES.MPD for each plot using the packages ‘ape’ (Paradis and Schliep 2019) and ‘picante’ (Kembel et al. 2010) in R (R Core Team 2022), we obtained a gradient of plant phylogenetic distance, where positive values corresponded to communities overdispersed along the phylogeny with greater evolutionary distances, and negative values result from communities of species clustered in specific clades, with more recent ancestors.

To investigate the success of restoration plots in the recovery of dung beetle communities (Prediction 2), we collected dung beetles at nine study sites (three per habitat type) in June and August 2022: cattle pastures, restoration plots, and native forest. Pasture sites were delimited in a 15 m × 15 m area (to match the dimension of restoration plots) located within the active cattle foraging areas, at least 50 m from the edge of the forest and restoration plots described above, and 100 m from each other. The pasture sites had mostly gramineous and herbaceous species where cattle regularly forage. However, all livestock were excluded from this area 8 days prior to our sampling. For the restoration plots, we randomly selected three out of the 22 restoration plots described above and used the data from both samplings. Finally, the native forest sites were located within the neighboring forest fragment (ca. 18 ha) with at least 300 m distance from each other and with a minimum distance of 50 m from the pasture matrix.

### Dung beetle sampling

Dung beetles were collected with pitfall traps baited with 50 g of fresh cattle dung. Although cattle feces have been shown to attract fewer individuals and species of dung beetles compared to other baits (Amézquita and Favila 2010; González-Tokman et al. 2018; Mora-Aguilar et al. 2023), we used this bait because it effectively attracts a subset of species adapted to native dung (i.e., monkey dung; Amézquita and Favila 2010), and because cattle feces represent the primary food resource for dung beetles in productive livestock landscapes (Arellano et al. 2023). Traps were plastic containers (14.5 cm tall and 11.5 cm opening diameter), filled up to one-third of their capacity with salty and soapy water, and were buried flush to ground level. We placed a wire-supported plastic plate above each pitfall trap to hang the dung bait in a small mesh net and to protect each trap from rain, direct sun, or fallen debris.

We used five pitfall traps at each of the 28 study sites (22 restoration plots, 3 pasture plots, and 3 forest sites), which were ≥ 50 m apart to avoid interference among samples (Mora-Aguilar et al. 2023). In the restoration and pasture plots, one trap was placed at each corner (~ 2 m away from the edge of the plot) and one in the center. At the forest sites, traps were placed 7–10 m apart along a transect distanced at least 50 m from the edge of the fragment. The contents of each set of five pitfall traps within a site/plot were combined and considered as the sampling unit for that site. The traps were exposed for 48 h per sampling period and the baits were replaced after the first 24 h. All specimens were identified in the laboratory using keys, descriptions, and dung beetle collections of species from the Los Tuxtlas region.

### Functional diversity of dung beetles

To assess the functional diversity of dung beetles, we selected species with more than three collected individuals, measuring a set of functional traits for 12 species in total (Online Resources 1, 2). For each species, we calculated the mean values of the biomass, pronotum volume (area × height), foreleg and hindleg area, foreleg and hindleg length, and the ratio of hindleg and foreleg length. Body measurements were obtained using ImageJ v1.53t (Abràmoff et al. 2004). Furthermore, based on published data (Montes de Oca and Halffter 1998; Galante et al. 2003; Díaz et al. 2010; Rivera et al. 2022), we classified each species according to their food preferences (coprophagous or necrophagous), activity period (diurnal or nocturnal), and dung removal strategy (dweller, roller, or tunneller). Morphological traits are related to the ability of dung beetles to disperse seeds and dig dung (Nervo et al. 2014; Griffiths et al. 2015), and categorical traits represent the variation in resource manipulation and the temporal segregation of their functional activities (Slade et al. 2007; Manning et al. 2016).

We used the R package ‘mFD’ (Magneville et al. 2022) to calculate three components of functional alpha diversity: functional originality (FOri), functional specialization (FSpe), and functional dispersion (FDis). Functional originality indicates the uniqueness of the species in a community, and it is measured as the average pairwise distance between a species and its nearest neighbor within the trait morphospace (Mouillot et al. 2013). Functional specialization quantifies the degree of specialized trait combinations a species community possesses (Devictor et al. 2008), and it is computed as the weighted mean distance of species from the center of the trait space of the species pool (Mouillot et al. 2013). Functional dispersion determines the functional dissimilarity within a species community by measuring the mean distance of all species from the weighted centroid of the trait space (Laliberté and Legendre 2010; Pavoine et al. 2017). The functional distances between species were based on the Gower distance, as continuous and categorical traits were used. Furthermore, we used the minimal mean squared-deviation index (mSD) to select the number of PCoA axes used to calculate functional diversity metrics (Podani 1999; Maire et al. 2015). mSD values closer to 0 indicate a more accurate representation of the functional space based on the Gower distances.

### Phylogenetic diversity of dung beetle species

To assess the phylogenetic diversity of the collected dung beetles, we calculated the MPD and the mean nearest taxon distance (MNTD). The latter determines the pairwise phylogenetic distances between the closest relatives within a community (Webb et al. 2002). We reconstructed a phylogenetic tree (Online Resource 3) using all beetle species collected from the 28 study sites. MPD and MNTD were calculated for each dung beetle community using the ‘Picante’ package (Kembel et al. 2010) in R (R Core Team 2022). Information about the tree reconstruction details can be found in Online Resource 4.

### Null models

Phylogenetic and functional diversity are often positively related to species richness (Mouchet et al. 2010). In our study, we observed a positive relationship between plant MPD and plant species richness (Online Resource 5). Similarly, for dung beetles, we found a positive relationship between FDis and MPD with dung beetle species richness for data used to test Prediction 1 (Online Resource 5). For data used to test Prediction 2, the relationship between FSpe and dung beetle species richness was marginally significant (Online Resource 5). To account for these significant relationships and to provide measures of functional and phylogenetic diversity independent of species richness, we used the standardized effect size (SES) of MPD and FSpe (Mouchet et al. 2010). The SES was determined by calculating the difference between the observed diversity and the mean diversity of 999 randomly generated communities, divided by the standard deviation of the random values [SES = (observed diversity – X random diversity) / SD random diversity]. Random communities were generated using the independent-swap algorithm, which conserves observed species richness and occurrences at each site while randomizing species identity (Gotelli and Entsminger 2003). SES values close to or below -1.96 indicate that observed diversity is significantly lower than expected by chance, while values close to or above 1.96 indicate that observed diversity is significantly higher than expected by chance (Swenson 2014).

### Dung beetle biomass

To estimate dung beetle biomass (i.e., biomass of all individuals encountered at a specific site), we first dried ten individuals per species (or fewer, when there were < 10 captures per species) at 35 °C for 48 h to measure their dry weight on an analytical balance with a precision scale of 0.1 mg. This value was then multiplied by the abundance of each species. All biomass estimates for each species were added to calculate the total dung beetle biomass at each site.

### Data analysis

#### Prediction 1

To evaluate the influence of plant diversity on dung beetle communities in the 22 restoration plots, we pooled all individuals collected in each plot during both samplings. We then fitted generalized linear models (GLMs) using the R package ‘lme4’; (Bates et al. 2015) in R v 4.2.2 (R Core Team 2022) to assess how each predictor variable—plant species richness and phylogenetic diversity (Plant SES.MPD)—influenced the following beetle response variables: species richness, total biomass (mg; log_10_ transformed), FOri, FSpe, SES.FDis, SES.MPD, and MNTD. For beetle abundance, we fitted one model using the total abundance of dung beetles (Scarabaeinae + Aphodiinae) as a response variable, as well as two separate models for the abundances of Scarabaeinae and Aphodiinae. We used a separate model for each combination of response and predictor variables and used different error distribution and link functions for each case (as specified below). *R*^2^ values were obtained with the R package ‘performance’ (Lüdecke et al. 2021).

#### Prediction 2

To assess whether restoration plots facilitated the recovery of dung beetle communities in terms of biomass, taxonomic, functional, and phylogenetic diversity, we first evaluated the sampling efficiency using the coverage estimator proposed by Chao and Jost (2012), implemented with the iNEXT online software (Chao et al. 2016). Then, we analyzed species richness, total biomass (mg; log_10_ transformed), FOri, SES.FSpe, FDis, MPD, and MNTD as response variables in GLMs, with habitat type (native forest, restoration plots, and cattle pasture) as the predictor variable. The influence of habitat type was determined with the Wald–Chi test, applied through the Anova function (R package ‘car’; Fox and Weisberg 2019). Post hoc comparisons were conducted using the emmeans function (R package ‘emmeans’; Lenth 2020), and the Bonferroni method to adjust P values for multiple comparisons.

For species richness and Scarabaeinae abundance, GLMs were adjusted with a Poisson error distribution and a log link function. When overdispersion of the variance was detected, such as for the abundance of dung beetles (Scarabaeinae + Aphodiinae) and the abundance of Aphodiinae, a negative binomial error structure with a log link function was applied. For all other response variables—total biomass, FOri, FSpe, SES.FDis, SES.MPD, and MNTD for Prediction 1, and total biomass, FOri, SES.FSpe, FDis, MPD, and MNTD for Prediction 2—GLMs were fitted using a Gaussian error distribution with an identity link function. Model suitability was evaluated by examining the standard residuals vs fitted values and visually inspecting the distribution of errors.

We performed a Moran’s *I* test using the ‘sp’ and ‘spdep’ R packages (Bivand 2022) to assess spatial autocorrelation for all dung beetle response variables (Online Resource 6). For functional and phylogenetic metrics, the Moran’s *I* test was applied only to the observed data, as the standardized effect size (SES) values are derived from null/randomized communities and do not reflect the actual spatial structure of these diversity metrics. Significant spatial structure was detected only for FDis values in Prediction 2 (Online Resource 6). However, because habitat type had no significant influence on this predictor variable, we did not apply any spatial correction.

## Results

### Dung beetles

We collected a total of 236 dung beetles from 13 species across the 22 restoration plots (Online Resource 2). The most abundant species were *Ataenius* aff. *crenulatus* (47%), *A.* sp. 2 (25%) and *Eurysternys mexicanus* (16%). For the nine sites used to test the success of restoration plots in recovering dung beetle communities, we sampled eight species at the three forest sites (104 individuals), eight species at the three restoration plots (32 individuals), and seven species at the three cattle pasture sites (52 individuals; Online Resource 2). The sampling efficiency was 100% in the native forest, 85% in the restoration plots, and 96% in the cattle pasture. The dominant species in the native forest were *Copris laeviceps*, *E. maya*, and *Ateuchus illaesum*, which together accounted for 79% of the total number of dung beetles sampled in this habitat. In the restoration plots, the dominant species were *A.* aff. *crenulatus*, *A.* sp. 2, and *E. mexicanus*, representing 84% of the total number of dung beetles found. Finally, the most abundant species in the cattle pasture were *A.* aff. *crenulatus* and *A.* sp. 2, representing 79% of the total dung beetles sampled in this habitat (Online Resource 2).

### Influence of plant species richness and phylogenetic diversity on the diversity and biomass of dung beetles

**Fig. 1:**
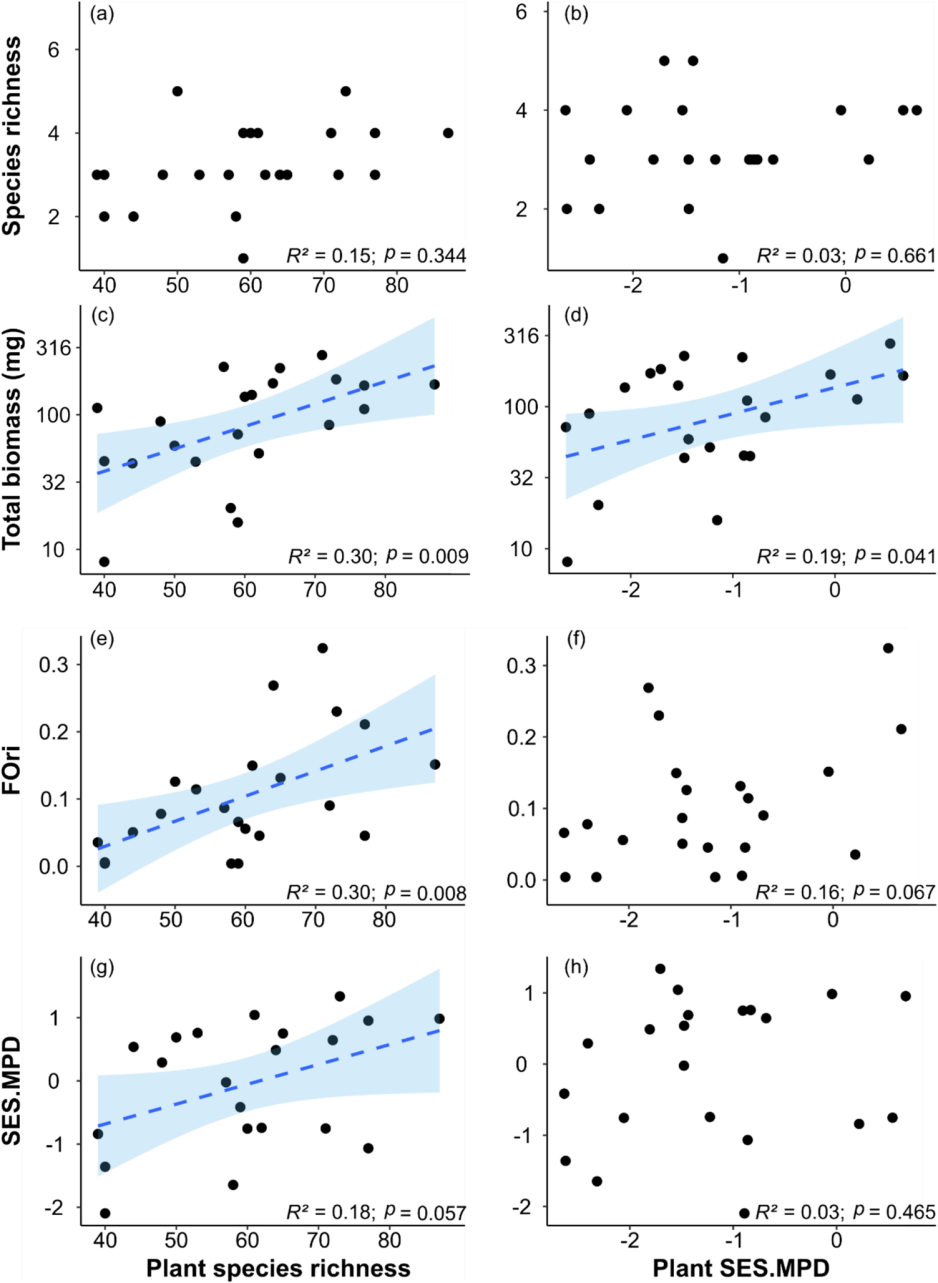
Relationships between dung beetle diversity and plant diversity in the 22 restoration plots (15 m × 15 m). Dung beetle metrics include (**a**, **b**) species richness, (**c**, **d**) total biomass, (**e**, **f**) functional originality (FOri), and (**g**, **h**) mean pairwise distance (SES.MPD). Plant diversity metrics are represented by plant species richness (left column) and plant phylogenetic diversity (SES mean pairwise distance; right column). The concave curves show the ± 95% confidence intervals of the model predictions

**Fig. 2:**
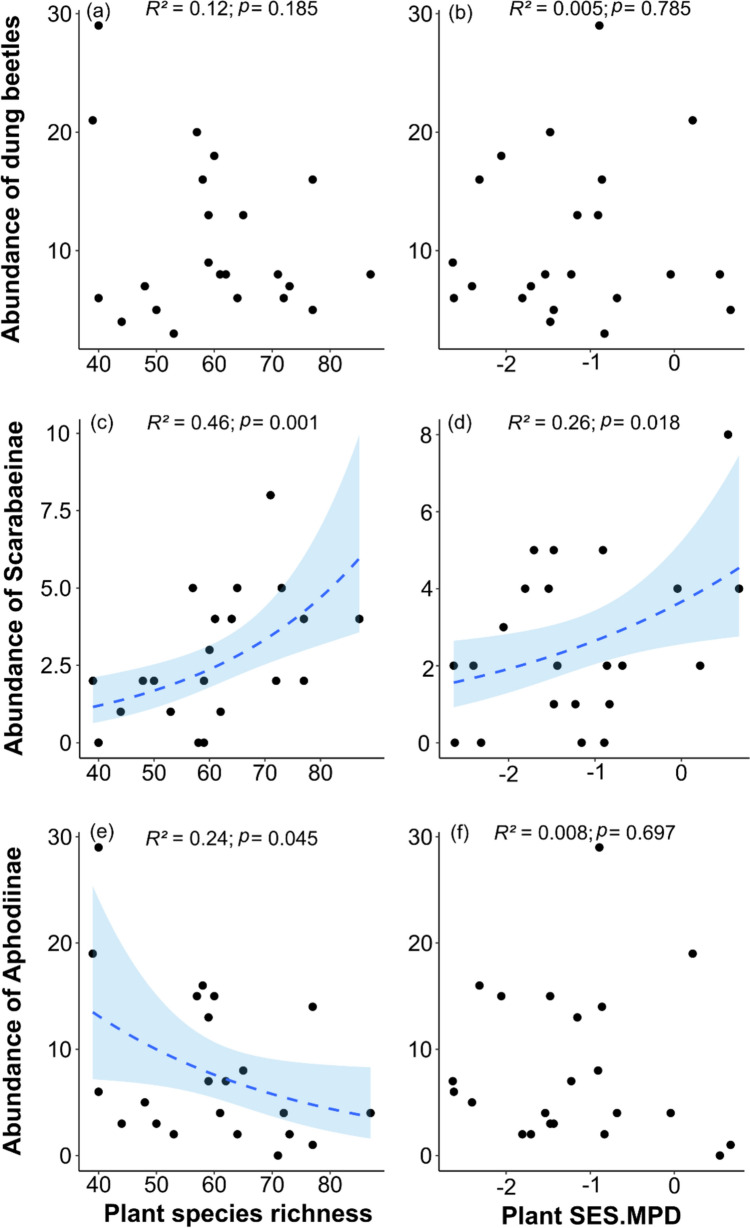
Relationships between (**a**, **b**) dung beetle abundance, (**c**, **d**) Scarabaeinae abundance, and (**e**, **f**) Aphodiinae abundance and two plant diversity metrics: plant species richness (left column) and plant phylogenetic diversity (SES mean pairwise distance; right column) in the 22 restoration plots (15 m × 15 m). GLMs with a negative binomial error structure (log link function) were used for the abundance of dung beetles (Scarabaeinae + Aphodiinae) and the abundance of Aphodiinae due to variance overdispersion, while a Poisson error structure (log link function) was applied for Scarabaeinae abundance. Concave curves represent ± 95% confidence intervals of the model predictions

According to our prediction, we found that the total biomass and the FOri of all dung beetles, together with the abundance of Scarabaeinae, were positively related to the number of plant species (Table 1, Figs. 1, 2), whereas the abundance of Aphodiinae was negatively related to plant species richness (Table 1; Fig. 2). However, the relationship between dung beetle species richness, abundance, FSpe, SES.FDis, and the MNTD and plant species richness was rather small (*R*^*2*^ < 0.20) and not significant. In the case of dung beetle phylogenetic diversity (SES.MPD), plant richness had a marginally significant relationship (*P* = 0.057) (Table 1; Figs. 1, 2; Online Resource 7). We also found positive but relatively weak significant relationships between dung beetle total biomass and abundance of Scarabaeinae with plant phylogenetic diversity (*R*^*2*^ = 0.19 and 0.26, respectively; Table 2, Figs. 1, 2). In contrast, no significant relationships were detected between any of the other eight response variables of dung beetles and the plant phylogenetic diversity (Table 2, Figs. 1, 2; Online Resource 7). Finally, we found that higher values of total biomass of dung beetles were explained by an increase in dung beetle FOri (Online Resource 8a) and by a greater abundance of relatively large dung beetles (Online Resource 8b).

**Table 1:** Results of the GLMs that assess the relationships between the dung beetle response variables: species richness, abundance of dung beetles (Scarabaeinae + Aphodiinae), abundance of Scarabaeinae, abundance of Aphodiinae, log10 total biomass (mg), functional originality (FOri), functional specialization (FSpe), functional dispersion (SES.FDis), mean pairwise distance (SES.MPD), and mean nearest taxon distance (MNTD) and the number of plant species in the 22 restoration plots (15 m × 15 m) where dung beetles were sampled

	Estimate^a^	se^a^	*Z*/*t*	*P*^a^
Species richness	*R*^2^ = 0.15			
(Intercept)	0.641	0.579	1.107	0.268
Plant species richness	0.008	0.009	0.946	0.344
Abundance of dung beetles	*R*^2^ = 0.12			
(Intercept)	3.099	0.564	5.493	< 0.0001
Plant species richness	− 0.012	0.009	− 1.326	0.185
Abundance of Scarabaeinae	*R*^2^ = 0.46			
(Intercept)	− 1.188	0.696	− 1.706	0.088
Plant species richness	0.034	0.01	3.269	**0.001**
Abundance of Aphodiinae	*R*^2^ = 0.24			
(Intercept)	3.667	0.823	4.453	< 0.0001
Plant species richness	− 0.273	0.013	− 1.998	**0.045**
log_10_Total biomass	*R*^2^ = 0.30			
(Intercept)	0.913	0.352	2.591	0.017
Plant species richness	0.016	0.005	2.891	**0.009**
FOri	*R*^2^ = 0.30			
(Intercept)	− 0.119	0.078	− 1.526	0.142
Plant species richness	0.003	0.001	2.912	**0.008**
FSpe	*R*^2^ = 0.07			
(Intercept)	0.294	0.012	23.622	< 0.001
Plant species richness	0.0002	0.0002	1.265	0.22
SES.FDis	*R*^2^ = 0.08			
(Intercept)	− 1.631	0.653	− 2.495	0.021
Plant species richness	− 0.013	0.01	-1.308	0.206
SES.MPD	*R*^2^ = 0.18			
(Intercept)	− 1.938	0.95	− 2.039	0.055
Plant species richness	0.031	0.015	2.025	0.057
MNTD	*R*^2^ = 0.02			
(Intercept)	0.294	0.196	1.501	0.15
Plant species richness	0.001	0.003	0.598	0.557

^a^The Estimate and se columns show the estimate and standard error of the model

The *P* values are based on the *Z*-statistics for the Poisson and negative binomial models and on *t*-statistics for the Gaussian models. Significant predictors are in bold (*P* < 0.05). The *R*^2^ values are given for each model

**Table 2:** Results of the GLMs testing the relationships between the dung beetle response variables: species richness, abundance of dung beetles (Scarabaeinae + Aphodiinae), abundance of Scarabaeinae, abundance of Aphodiinae, log_10_ total biomass (mg), functional originality (FOri), functional specialization (FSpe), functional dispersion (SES.FDis), mean pairwise distance (SES.MPD), and mean nearest taxon distance (MNTD) and the mean pairwise phylogenetic diversity (SES.MPD) of plants in the 22 restoration plots (15 m × 15 m) where dung beetles were sampled

	Estimate^a^	se^a^	Z/t	*P*^a^
Number of species	*R*^2^ = 0.03			
(Intercept)	1.237	0.188	6.552	< 0.001
Plant SES.MPD	0.055	0.126	0.438	0.661
Abundance of dung beetles	*R*^2^ = 0.005			
(Intercept)	2.415	0.200	12.06	< 0.001
Plant SES.MPD	0.035	0.131	0.273	0.785
Abundance of Scarabaeinae	*R*^2^ = 0.26			
(Intercept)	1.296	0.182	7.111	< 0.001
Plant SES.MPD	0.323	0.136	2.366	**0.018**
Abundance of Aphodiinae	*R*^2^ = 0.008			
(Intercept)	1.999	0.308	6.478	< 0.001
Plant SES.MPD	− 0.078	0.201	− 0.390	0.697
log_10_ Total biomass	*R*^2^ = 0.19			
(Intercept)	2.134	0.129	16.48	< 0.001
Plant SES.MPD	**0.184**	**0.084**	**2.177**	**0.041**
FOri	*R*^2^ = 0.16			
(Intercept)	0.148	0.029	5.054	< 0.001
Plant SES.MPD	0.037	0.019	1.934	0.067
FSpe	*R*^2^ = 0.07			
(Intercept)	0.314	0.004	73.46	< 0.001
Plant SES.MPD	0.003	0.002	1.257	0.223
SES.FDis	*R*^2^ = 0.04			
(Intercept)	− 2.627	0.231	− 11.35	< 0.001
Plant SES.MPD	− 0.132	0.15	− 0.882	0.389
SES.MPD	*R*^2^ = 0.03			
(Intercept)	0.151	0.357	0.423	0.677
Plant SES.MPD	0.172	0.231	0.745	0.465
MNTD	*R*^2^ = < 0.01			
(Intercept)	0.411	0.068	5.992	< 0.001
Plant SES.MPD	0.001	0.044	0.024	0.981

^a^The Estimate and se columns show the estimate and standard error of the model

The *P* values are based on the *Z*-statistics for the Poisson and negative binomial models and on *t*-statistics for the Gaussian models. Significant predictors are in bold (*P* < 0.05). The *R*^2^ values are given for each model

### Influence of restoration plots on the diversity and biomass of dung beetles

**Fig. 3:**
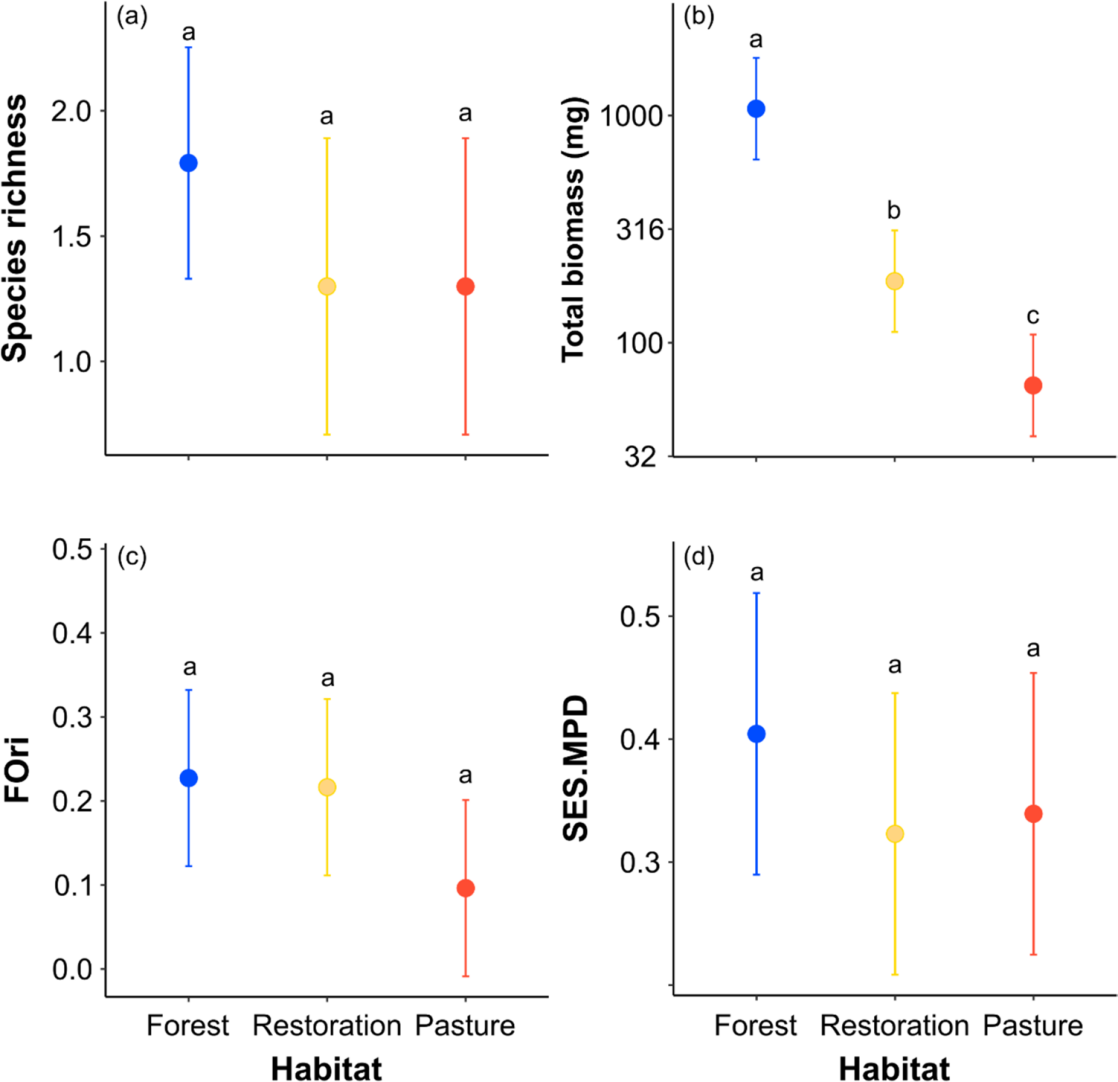
Mean ± 95% confidence intervals for (**a**) species richness, (**b**) total biomass, (**c**) functional originality (FOri), and (**d**) SES mean pairwise distance (MPD) of dung beetle communities sampled in the different habitat types: Native forest (blue), restoration plots (yellow), and cattle pasture (orange). Different letters indicate significant differences (*P* < 0.05) among habitat types based on pairwise Bonferroni correction for multiple comparisons

The total biomass of dung beetles was six times greater in the native forest than in the restoration plots and 16 times greater than in the cattle pastures (Fig. 3b). Moreover, as expected, beetle total biomass was three times greater in the restoration plots than in the cattle pastures (*χ*^*2*^ = 90.48, *df* = 2, *P* < 0.001; Fig. 3b). The species richness (*χ*^*2*^ = 2.339, *df* = 2, *P* = 0.310; Fig. 3a), FOri (*χ*^*2*^ = 3.686, *df* = 2, *P* = 0.158; Fig. 3c), SES.FSpe (*χ*^*2*^ = 4.374, *df* = 2, *P* = 0.112; Online Resource 9a), FDis (*χ2* = 0.855, *df* = 2, *P* = 0.652; Online Resource 9b), SES.MPD (*χ2* = 1.083, *df* = 2, *P* = 0.581; Fig. 3d), and MNTD of dung beetles (*χ2* = 0.052, df = 2, *P* = 0.973) were similar across habitat types (Online Resource 9c).

## Discussion

This study provides empirical evidence that some diversity components of dung beetle communities can be influenced by plant species richness and phylogenetic diversity, despite the lack of a direct trophic relationship between both groups. However, the total biomass of dung beetles and the abundance of Scarabaeinae were the only metrics influenced by both plant diversity metrics. Our short-term assessment of dung beetle responses to restoration plots offers insights into biodiversity recovery in productive cattle systems, highlighting the relevance of plant species selection in restoration programs. Specifically, local plant communities with higher species richness and evolutionary distance seem to enhance such recovery.

A positive relationship between plant species richness and different diversity metrics of arthropod communities has been previously reported for different animal guilds, including herbivores (Zhang et al. 2017; Staab et al. 2021), predators (Dinnage et al. 2012), parasitoids (Salazar et al. 2016), leaf litter ants (Skarbek et al. 2020), and dung beetles (Menéndez et al. 2024). Although these findings are not surprising for the first three cases (due to the trophic relationship between host plants, herbivores, and their predators), the influence of plant communities on litter ants and dung beetles is remarkable, given that they do not consume plant tissues. In both cases, habitat heterogeneity, linked to increased litter input, can explain the positive influence of tree diversity on the abundance and species richness of these guilds (Skarbek et al. 2020; Menéndez et al. 2024). Thus, we hypothesize that our restoration plots had more complex habitats (i.e., more niches associated with the structural complexity of vegetation and leaf litter composition) relative to what was available in livestock pastures, which in turn favored the recovery of dung beetle communities.

We found that the functional originality and the standardized effect size of mean phylogenetic pairwise distances among dung beetle species were positively related to plant species richness. This suggests that plant communities with fewer plant species are likely to have environmental filters and/or limited ecological niches, which restrict the coexistence of dung beetle species from evolutionary distant clades with different functional attributes. In contrast, plant communities with higher plant diversity may increase habitat heterogeneity, promoting the coexistence of dung beetle species from different phylogenetic origins and ecological niches. The greater availability of ecological niches in these plant communities could be linked to the increase in canopy cover heterogeneity, which, in turn, can modify microclimatic conditions such as temperature and humidity (Fornoff et al. 2021). These variables are known to play a key role in the assembly of dung beetle communities after active restoration in tropical areas (Audino et al. 2017). Therefore, restoration plots with high plant species richness, by promoting forest-like environments, could play an outstanding role in the recovery of functionally diverse dung beetle assemblages. Additionally, because mammal diversity can increase in productive landscapes surrounded by natural habitats (Piña et al. 2019), and because changes in mammal species and abundance can impact dung beetle communities (Raine and Slade 2019), it is possible that tree species richness indirectly influenced dung beetle recovery by providing more dung resources. Further investigation is required to assess mammal communities in these restoration plots.

We found that the abundance of Aphodiinae beetles was negatively related to plant species richness. This could be partly explained by the heliophilous habits of *Ataenius* species (the only Aphodiinae genus in our samples), which are commonly associated with pastures and cattle dung (Galante et al. 2003; Díaz et al. 2010). Consequently, habitats with less vegetation and/or lower leaf litter heterogeneity could favor the persistence of this group. In contrast, the positive relationship between the abundance of Scarabaeinae beetles and plant species and phylogenetic diversity could be explained by the preference of species within this group for forest-like microhabitats (Audino et al. 2017). In turn, these contrasting differences in habitat preferences might explain the negative relationship between the abundance of Aphodiinae and Scarabaeinae beetles (Online Resource 10). In addition, because Scarabaeinae beetles are more effective competitors for food resources in tropical climates (Hanski and Cambefort 1991), some species within this group (e.g., *Eurysternus*) may have been able to displace *Ataenius* in heliophilous environments, as they both are endocoprid beetles within cattle dung.

Our findings suggest that the presence of larger-bodied and functionally distinct dung beetle species was promoted by plant communities with greater phylogenetic divergence; thereby, these plant communities enhanced the functional diversity of beetle communities. Such an influence could be linked to the positive relationship between plant phylogenetic diversity and litter decomposition rates (Xiao et al. 2020), which influences dung beetles (Viegas et al. 2014; da Silva and Hernández 2016). For instance, previous studies have shown that the accumulation of leaf litter can negatively affect the nesting behavior of roller beetles (Nichols et al. 2013). Hence, plots with more diverse plant communities and increased litter decomposition rates are likely to have greater nesting activity of some dung beetle species, which might explain the greater abundance of Scarabaeinae beetles in plant communities with greater phylogenetic diversity. However, future studies are needed to identify the causal mechanisms through which plant phylogenetic diversity may influence dung beetle nesting behavior and biomass.

The total biomass of dung beetles was greater in restoration plots compared to cattle pasture sites. This finding contrasts with a previous study conducted in an active cattle pasture in the Los Tuxtlas region, which reported similar biomass levels between restoration plots and pastures (González-Tokman et al. 2018). These differences may be due to interannual variation and habitat specificity of dung beetles, leading to different conclusions depending on the sampling year (Beiroz et al. 2017). For example, *Eurysternus mexicanus* accounted for 34% of the dung beetles collected in our study, a species primarily associated with forested sites in the region (Salomão et al. 2020; Rivera et al. 2021), compared to only 2% reported by González-Tokman et al. (2018). Therefore, long-term studies are needed to better understand the impacts of restoration plots on dung beetle communities in relation to interannual and climatic variability.

In conclusion, our findings show that restoration plots with high plant species richness and high plant phylogenetic diversity can favor the recovery of functionally diverse dung beetle communities in the short term within a relatively small area. This supports the idea that, by recovering local environmental variables and potentially increasing micro-niche diversity, active restoration plots can represent ‘habitat extensions’ (e.g., Carvalho et al. 2023) for sensitive dung beetle species in forested areas surrounding productive landscapes. However, our results should be interpreted with caution, as our study lacks replicated plots across different landscapes (Howe and Martínez-Garza 2014) and relies on synthetic plant communities. Hence, further research is needed to generalize our findings to other agricultural and cattle ranching contexts. Finally, our study highlights the potential of restoration plots within active cattle pastures to play a key role in recovering dung beetle biomass, which in turn can enhance ecosystem functions (Ratoni et al. 2023; Rivera et al. 2024, Menéndez et al. 2024) and improve livestock productivity through the processing of organic matter into the soil (Lopez-Collado et al. 2017).

## Supplementary Information

Below is the link to the electronic supplementary material.

Supplementary file1 (DOCX 537 KB)

## Data Availability

The data sets generated during the current study are available from the corresponding author on reasonable request.
