# Supplementary material for: Plant species richness and phylogenetic diversity can favor the recovery of dung beetle communities in ecological restoration plots
Source: Oecologia. 2025 Feb 1;207(2):29. doi: 10.1007/s00442-025-05666-8 (PMC11787278; doi:10.1007/s00442-025-05666-8)
Supplement: Supplementary file 1 — Supplementary file1 (DOCX 537 KB) [file 442_2025_5666_MOESM1_ESM.docx]

**Electronic Supplementary Material**

Manuscript title: Plant species richness and phylogenetic diversity can favor the recovery of dung beetle communities in ecological restoration plots

Authors: Lina Adonay Urrea-Galeano, Rocío Santos-Gally, José D. Rivera-Duarte, Alfonso Díaz Rojas, Karina Boege

Corresponding author: Karina Boege; Email: [kboege@unam.mx](mailto:kboege@unam.mx)

**Online Resource 1.** Species traits used for the analysis of functional diversity. Body measurements: mean biomass (mBM), mean pronotum volume (mPV), mean foreleg area (mFLA), mean hind leg area (mHLA), and hind leg:foreleg length ratio (HLL/FLL) were calculated from values obtained through ImageJ. Information on food preference (FP), activity period (AP), and functional group (FG) is from Montes de Oca & Halffter (1998), Galante et al. 2003, Díaz et al. (2010), Rivera et al. (2022). See the main text for references.

| Species | mBM  (mg) | mPV (mm^3^) | MFLA (mm^2^) | mHLA  (mm^2^) | HLL/FLL | FP | AP | FG |
| --- | --- | --- | --- | --- | --- | --- | --- | --- |
| *Ataenius* aff. *crenulatus* | 1.220 | 1.522 | 0.376 | 0.403 | 1.343 | Copro | Nocturnal | Dweller |
| *Ataenius* aff. *sculptor* | 3.483 | 3.743 | 0.618 | 0.646 | 1.342 | Copro | Nocturnal | Dweller |
| *Ataenius* sp. 2 | 1.980 | 1.195 | 0.224 | 0.297 | 1.335 | Copro | Nocturnal | Dweller |
| *Ateuchus illaesum* | 8.700 | 12.367 | 1.173 | 1.233 | 1.154 | Copro | Nocturnal | Tunneller |
| *Canthidium centrale* | 14.750 | 31.860 | 2.370 | 3.028 | 1.294 | Copro | Nocturnal | Tunneller |
| *Canthon cyanellus cyanellus* | 22.200 | 41.909 | 3.388 | 3.499 | 1.322 | Necro | Diurnal | Roller |
| *Canthon euryscelis* | 5.340 | 12.324 | 1.385 | 1.753 | 1.384 | Copro | Diurnal | Roller |
| *Copris laeviceps* | 37.660 | 49.669 | 3.461 | 3.563 | 1.152 | Copro | Nocturnal | Tunneller |
| *Digitonthophagus gazella* | 30.057 | 69.946 | 4.556 | 3.705 | 0.867 | Copro | Diurnal | Tunneller |
| *Euoniticellus intermedius* | 7.267 | 22.965 | 1.884 | 2.114 | 1.158 | Copro | Diurnal | Tunneller |
| *Eurysternus maya* | 46.140 | 110.181 | 4.554 | 6.275 | 1.578 | Copro | Nocturnal | Dweller |
| *Eurysternus mexicanus* | 41.650 | 66.978 | 4.581 | 4.930 | 1.314 | Copro | Diurnal | Dweller |

**Online Resource 2.** Total number of individuals for each species of dung beetles (Scarabaeidae: Scarabaeinae and Aphodiinae) captured in pitfall traps baited with 50 g cow dung in three habitat types: native forest (F), restoration plots (R_all_ and R_3_), and cattle pasture (P). The R_all_ column shows the dung beetles captured in all 22 plots used for analyses of Prediction 1, while the R_3_ column shows the subset of dung beetles used for analyses of Prediction 2. The average values for biomass and body length of dung beetles are shown in the last two columns. Pitfall traps were deployed on two temporal occasions (June and August, 2022).

| Species | June 2022 | | | |  | August 2022 | | | | Biomass (mg) | Body length (mm) |
| --- | --- | --- | --- | --- | --- | --- | --- | --- | --- | --- | --- |
|  | Habitat | | | |  | Habitat | | | |  |  |
|  | F | R_all_ | R_3_ | P |  | F | R_all_ | R_3_ | P |  |  |
| *Ataenius* aff. *crenulatus*^a^ *Schmidt, 1910* | 0 | 1 | 0 | 8 |  | 0 | 111 | 9 | 24 | 1.220 | 3.78 |
| *Ataenius* aff. *sculptor*^a^ *Harold, 1868* | 0 | 0 | 0 | 0 |  | 0 | 5 | 0 | 3 | 3.483 | 5.18 |
| *Ataenius sp. 2*^a^ | 2 | 12 | 2 | 0 |  | 0 | 48 | 5 | 9 | 1.980 | 3.42 |
| *Ataenius sp. 3* | 0 | 0 | 0 | 1 |  | 0 | 1 | 1 | 0 | 3.100 | 5.44 |
| *Ataenius sp. 4* | 0 | 0 | 0 | 0 |  | 0 | 1 | 0 | 0 | 0.800 | 3.47 |
| *Ateuchus illaesum*^a^ *(Harold, 1868)* | 4 | 1 | 0 | 0 |  | 10 | 0 | 0 | 0 | 8.700 | 5.23 |
| *Canthidium centrale*^a^ *Boucomont, 1928* | 0 | 0 | 0 | 0 |  | 4 | 0 | 0 | 0 | 14.750 | 7.43 |
| *Canthidium pseudoperceptibile Kohlmann & Solis, 2006* | 0 | 0 | 0 | 0 |  | 2 | 0 | 0 | 0 | 0.400 | 4.23 |
| *Canthon cyanellus cyanellus*^a^ *Leconte 1859* | 0 | 0 | 0 | 0 |  | 0 | 2 | 0 | 1 | 22.200 | 7.47 |
| *Canthon euryscelis*^a^ *Bates, 1887* | 1 | 0 | 0 | 0 |  | 3 | 1 | 1 | 0 | 5.340 | 4.91 |
| *Copris laeviceps*^a^ *Harold, 1869* | 4 | 1 | 0 | 0 |  | 46 | 7 | 0 | 0 | 37.660 | 9.31 |
| *Digitonthophagus gazella*^a^** (Fabricius, 1787)* | 0 | 1 | 1 | 0 |  | 0 | 3 | 0 | 3 | 30.057 | 8.24 |
| *Euoniticellus intermedius*^a^** Reiche, 1849* | 0 | 0 | 0 | 1 |  | 0 | 0 | 0 | 2 | 7.267 | 6.18 |
| *Eurysternus maya*^a^ *Génier, 2009* | 1 | 0 | 0 | 0 |  | 17 | 2 | 1 | 0 | 46.140 | 11.29 |
| *Eurysternus mexicanus*^a^ *Harold, 1869* | 3 | 6 | 2 | 0 |  | 7 | 32 | 9 | 0 | 41.650 | 10.37 |
| *Onthophagus batesi Howden & Cartwright, 1963* | 0 | 1 | 1 | 0 |  | 0 | 0 | 0 | 0 | 18.100 | 6.38 |
| Total number of individuals | 15 | 23 | 6 | 10 |  | 89 | 213 | 26 | 42 |  |  |
| Total number of species | 6 | 7 | 4 | 3 |  | 7 | 11 | 6 | 6 |  |  |

^a^ species used for functional and phylogenetic analyses

*Introduced species

**
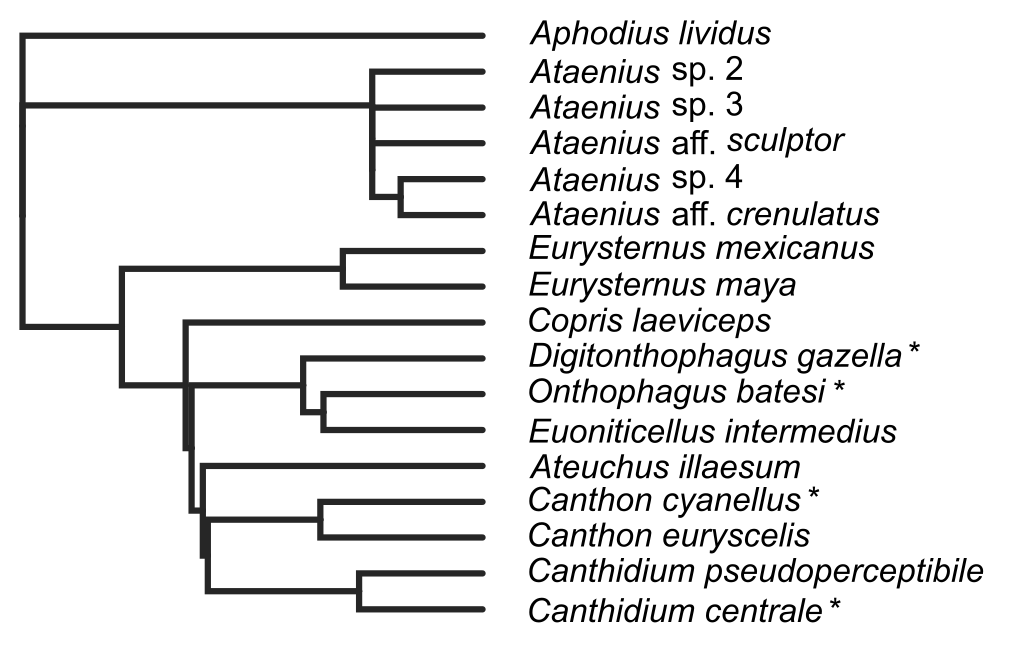
**

**Online Resource 3.** Reconstructed tree used to calculate the phylogenetic diversity of dung beetles. See Online Resource 4 (below) for further details regarding the phylogenetic reconstruction. GeneBank sequences were used for species marked with asterisks.

**Online Resource 4.** Details on how the phylogenetic tree of dung beetles was reconstructed.

We mixed eight phylogenetically informative markers for more than 500 samples in 11 genera from the data set of [Tarasov and Dimitrov (2016)](https://www.zotero.org/google-docs/?JCDjdH), with two markers (COI and 18S ribosomal RNA gene) from GenBank for three specimens of *Ataenius* (EF487634.1, EF487638.1, and EF487637.1), *Canthon cyanellus* (KX807690.1), *Canthidium centrale* (JQ972538.1), and *Onthophagus batesi* (EF656689.1). Alignments were made in Muscle v.3.8.31 [(Edgar 2004)](https://www.zotero.org/google-docs/?xJ7wEv) with manual refinement in PhyDE v.0.9971 [(Müller et al. 2006)](https://www.zotero.org/google-docs/?X8tIbh). An initial total tree was reconstructed in IQtree v.2.2.0.3 [(Minh et al. 2020)](https://www.zotero.org/google-docs/?lG19eJ) using multiple individuals per genera to avoid long-branch attraction in some particular clades due to some missing genes in our final alignment. We ran 1000 ultra-fast bootstrap [(Hoang et al. 2018)](https://www.zotero.org/google-docs/?ivgGLX) replicates to assess support values. For species in our sampling without sequences in the data set or GenBank, we selected a close relative species in the same genus as a representative for the final phylogeny following [(Rivera et al. 2022)](https://www.zotero.org/google-docs/?8XVqUG). Then, we pruned the final tree to retain the samples of interest for further analysis of phylogenetic diversity.

**References**

[Edgar RC (2004) MUSCLE: multiple sequence alignment with high accuracy and high throughput. Nucleic Acids Res 32:1792–1797. https://doi.org/10.1093/nar/gkh340](https://www.zotero.org/google-docs/?XiYh2g)

[Hoang DT, Chernomor O, von Haeseler A, et al (2018) UFBoot2: Improving the ultrafast bootstrap approximation. Mol Biol Evol 35:518–522. https://doi.org/10.1093/molbev/msx281](https://www.zotero.org/google-docs/?XiYh2g)

[Minh BQ, Schmidt HA, Chernomor O, et al (2020) IQ-TREE 2: New models and efficient methods for phylogenetic inference in the genomic era. Mol Biol Evol 37:1530–1534. https://doi.org/10.1093/molbev/msaa015](https://www.zotero.org/google-docs/?XiYh2g)

[Müller J, Müller K, Neinhuis C, Quandt D (2006) PhyDE-Phylogenetic Data Editor. http://www.phyde.de/. Accessed 22 Jun 2023](https://www.zotero.org/google-docs/?XiYh2g)

[Rivera JD, Espinosa de los Monteros A, da Silva PG, Favila ME (2022) Dung beetles maintain phylogenetic divergence but functional convergence across a highly fragmented tropical landscape. J Appl Ecol 59:1781–1791. https://doi.org/10.1111/1365-2664.14185](https://www.zotero.org/google-docs/?XiYh2g)

**Online Resource 5.** Results of GLMs that evaluated the relationship between plant phylogenetic diversity and plant species richness in the 22 restoration plots and, in the case of dung beetle communities, the relationships between the functional diversity (FOri: functional originality; FSpe: functional specialty; FDis: functional dispersion), the phylogenetic diversity (MPD: mean pairwise distance; MNTD: mean nearest taxon distance) and their respective species richness (predictor variable). An independent model was fitted for each response variable considering a Gamma error distribution. Significant predictors at p < 0.05 are in bold. The Est. and se columns show the estimate and standard error of the model. The P-values are based on the t-statistics. The R² values are given for each model.

|  |  | Est. | se | t | *p* |
| --- | --- | --- | --- | --- | --- |
| Plants | **MPD** | R² = 0.22 |  |  |  |
|  | (Intercept) | 230.66 | 2.73 | 84.61 | <0.0001 |
|  | Plant richness | 0.107 | 0.04 | 2.417 | **0.025** |
| Restoration plots (Prediction 1) | **FOri** | R² = 0.11 |  |  |  |
|  | (Intercept) | 0.004 | 0.07 | 0.063 | 0.951 |
|  | Beetle richness | 0.032 | 0.02 | 1.571 | 0.132 |
|  | **FSpe** | R² = 0.11 |  |  |  |
|  | (Intercept) | 0.29 | 0.01 | 32.45 | <0.0001 |
|  | Beetle richness | 0.004 | 0 | 1.593 | 0.127 |
|  | **FDis** | R² = 0.52 |  |  |  |
|  | (Intercept) | -0.069 | 0.05 | -1.27 | 0.218 |
|  | Beetle richness | 0.079 | 0.02 | 4.651 | **0.0001** |
|  | **MPD** | R² = 0.50 |  |  |  |
|  | (Intercept) | -0.081 | 0.1 | -0.84 | 0.41 |
|  | Beetle richness | 0.121 | 0.03 | 4.338 | **0.0003** |
|  | **MNTD** | R² = 0.005 |  |  |  |
|  | (Intercept) | 0.45 | 0.17 | 2.648 | 0.015 |
|  | Beetle richness | -0.015 | 0.05 | -0.3 | 0.768 |
| Native forest, Restoration plots, and Pasture  (Prediction 2) | **FOri** | R² = 0.002 |  |  |  |
|  | (Intercept) | 0.19 | 0.1 | 1.829 | 0.11 |
|  | Beetle richness | -0.002 | 0.02 | -0.11 | 0.918 |
|  | **FSpe** | R² = 0.43 |  |  |  |
|  | (Intercept) | 0.34 | 0.02 | 21.45 | <0.0001 |
|  | Beetle richness | -0.008 | 0 | -2.33 | 0.052 |
|  | **FDis** | R² = 0.026 |  |  |  |
|  | (Intercept) | 0.234 | 0.08 | 3.058 | 0.018 |
|  | Beetle richness | -0.007 | 0.02 | -0.43 | 0.679 |
|  | **MPD** | R² = 0.026 |  |  |  |
|  | (Intercept) | 0.31 | 0.11 | 2.825 | 0.025 |
|  | Beetle richness | 0.01 | 0.02 | 0.431 | 0.679 |
|  | **MNTD** | R² = 0.24 |  |  |  |
|  | (Intercept) | 0.704 | 0.2 | 3.598 | 0.008 |
|  | Beetle richness | -0.063 | 0.04 | -1.51 | 0.174 |

**Online Resource 6.** Moran's *I* spatial analysis for the beetle response variables of Prediction 1 and Prediction 2. Significant *p* vales (< 0.05) are in bold.

|  | Response variable | Observed value | Expected value | ± sd | *p* |
| --- | --- | --- | --- | --- | --- |
| Restoration plots (Prediction 1) | Species richness | 0.08 | -0.047 | 0.011 | 0.115 |
|  | Abundance of dung beetles | -0.201 | -0.047 | 0.011 | 0.928 |
|  | Abundance of Scarabaeinae | 0.07 | -0.047 | 0.011 | 0.133 |
|  | Abundance of Aphodiinae | -0.144 | -0.047 | 0.01 | 0.824 |
|  | log_10_ Total biomass | 0.033 | -0.047 | 0.011 | 0.224 |
|  | Functional originality | -0.041 | -0.047 | 0.011 | 0.477 |
|  | Functional specialty | 0.016 | -0.047 | 0.01 | 0.264 |
|  | Functional dispersion | 0.058 | -0.047 | 0.011 | 0.165 |
|  | Mean pairwise distance | -0.09 | -0.05 | 0.012 | 0.639 |
|  | Mean nearest taxon distance | -0.127 | -0.05 | 0.012 | 0.76 |
| Native forest, Restoration plots, and Pasture  (Prediction 2) | Species richness | 0.101 | -0.125 | 0.028 | 0.445 |
|  | log_10_ Total biomass | -0.195 | -0.125 | 0.032 | 0.653 |
|  | Functional originality | -0.186 | -0.125 | 0.031 | 0.634 |
|  | Functional specialty | 0.035 | -0.125 | 0.029 | 0.175 |
|  | Functional dispersion | 0.163 | -0.125 | 0.029 | **0.046** |
|  | Mean pairwise distance | 0.086 | -0.125 | 0.028 | 0.106 |
|  | Mean nearest taxon distance | -0.202 | -0.125 | 0.026 | 0.684 |


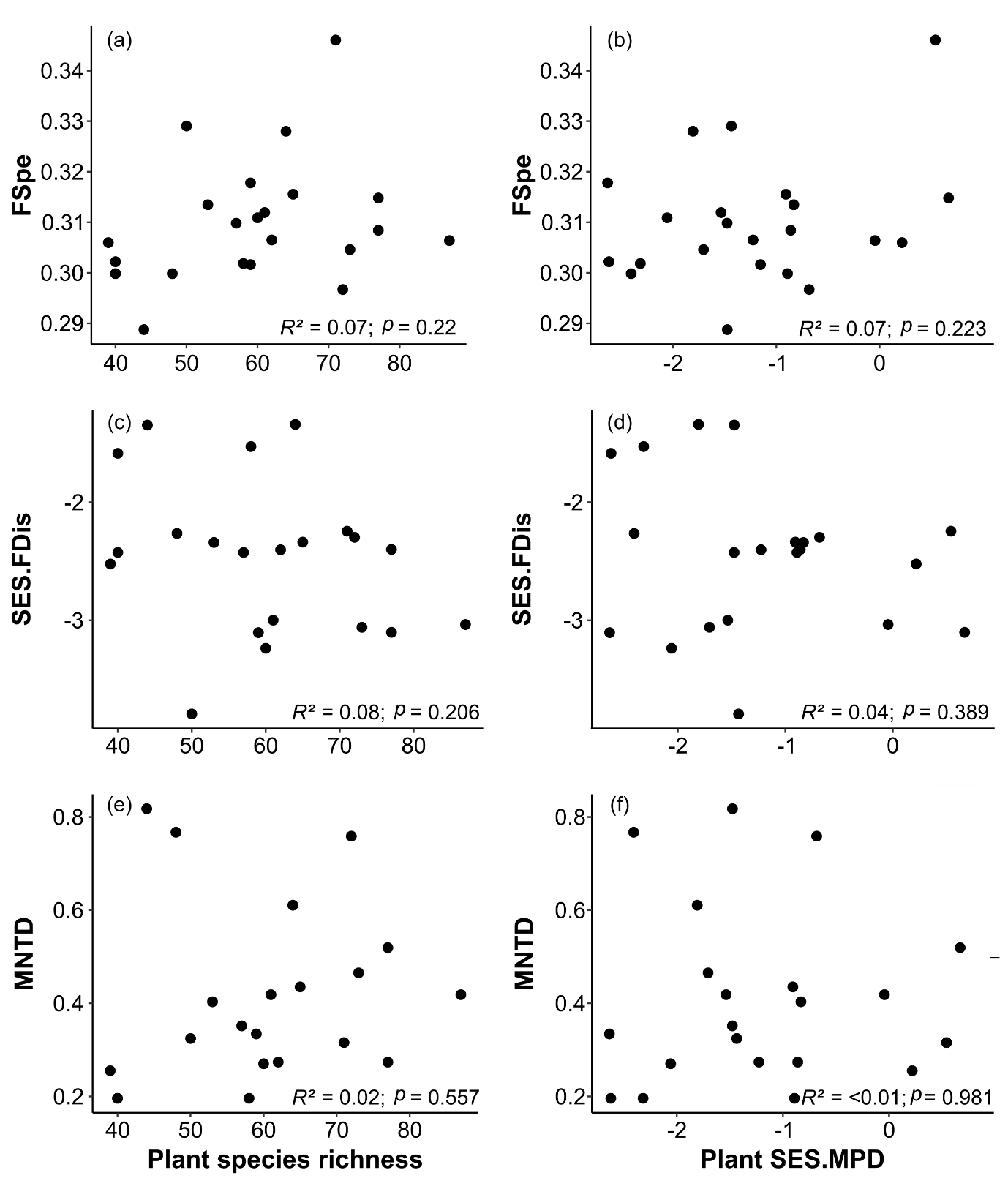


**Online Resource 7.** Relationships between (a,b) the functional specialization (FSpe), (c,d) the functional dispersion (SES.FDis), and (e,f) the mean nearest taxon distance (MNTD) of the dung beetle communities and plant species richness (left column) or plant phylogenetic diversity (SES.MPD; right column) in the 22 restoration plots (15 m × 15 m).


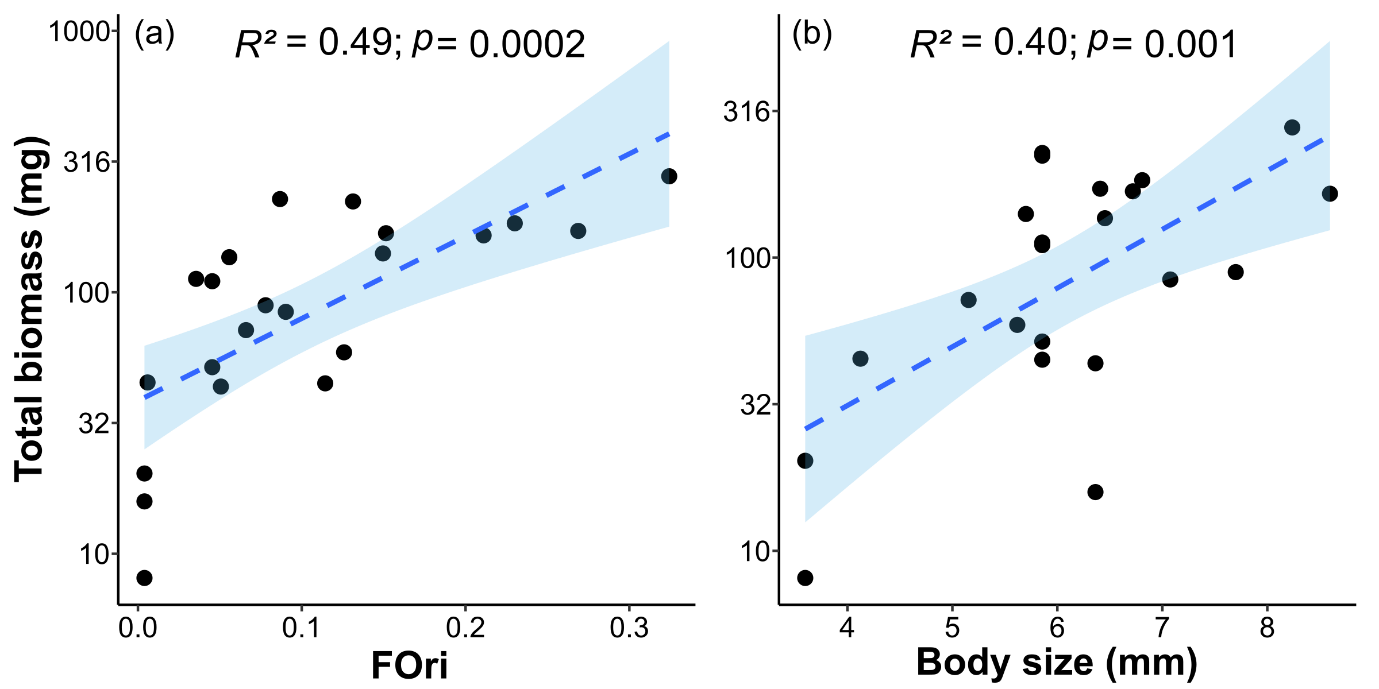


**Online Resource 8.** Total biomass of dung beetles as a function of their (a) functional originality (FOri) and (b) body size. The model was performed with log_10_-transformed values of the total biomass and used a gaussian error distribution for each model.


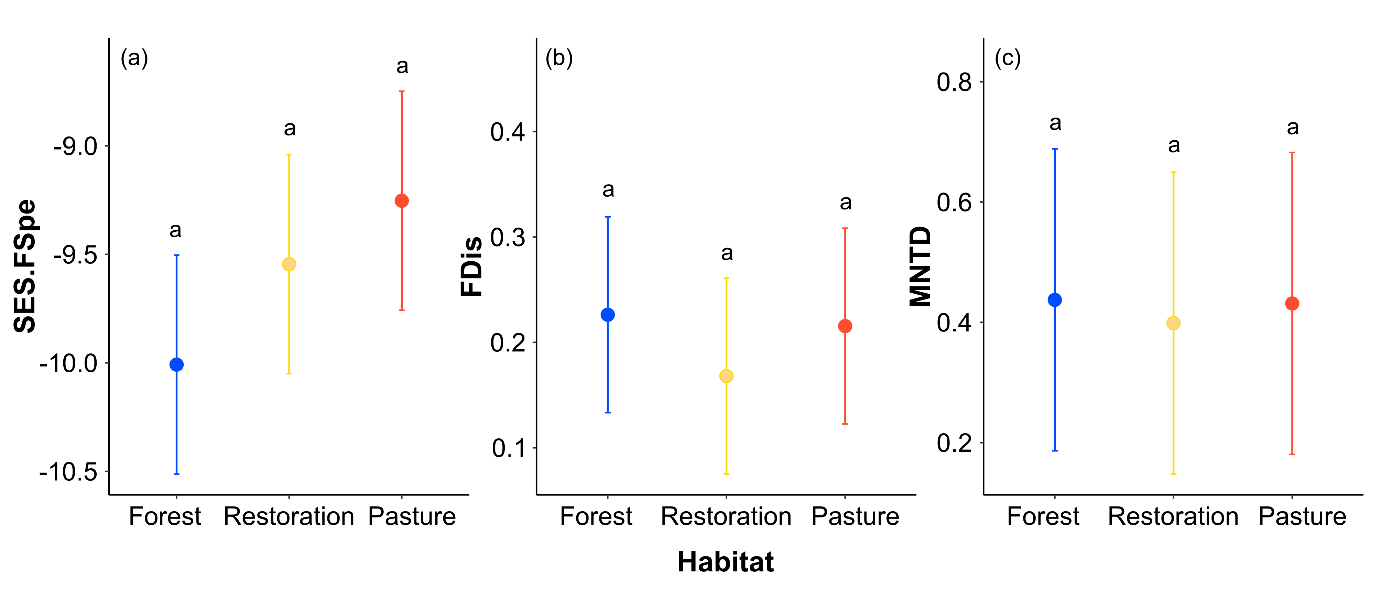


**Online Resource 9.** Mean ± 95% confidence intervals of (a) functional specialization (SES.FSpe), (b) functional dispersion (FDis), and (c) mean nearest taxon distance (MNTD) values of dung beetle communities in three habitat types: Native forest (blue), restoration plots (yellow) and cattle pastures (orange). Same letters indicate no significant differences (p > 0.05).


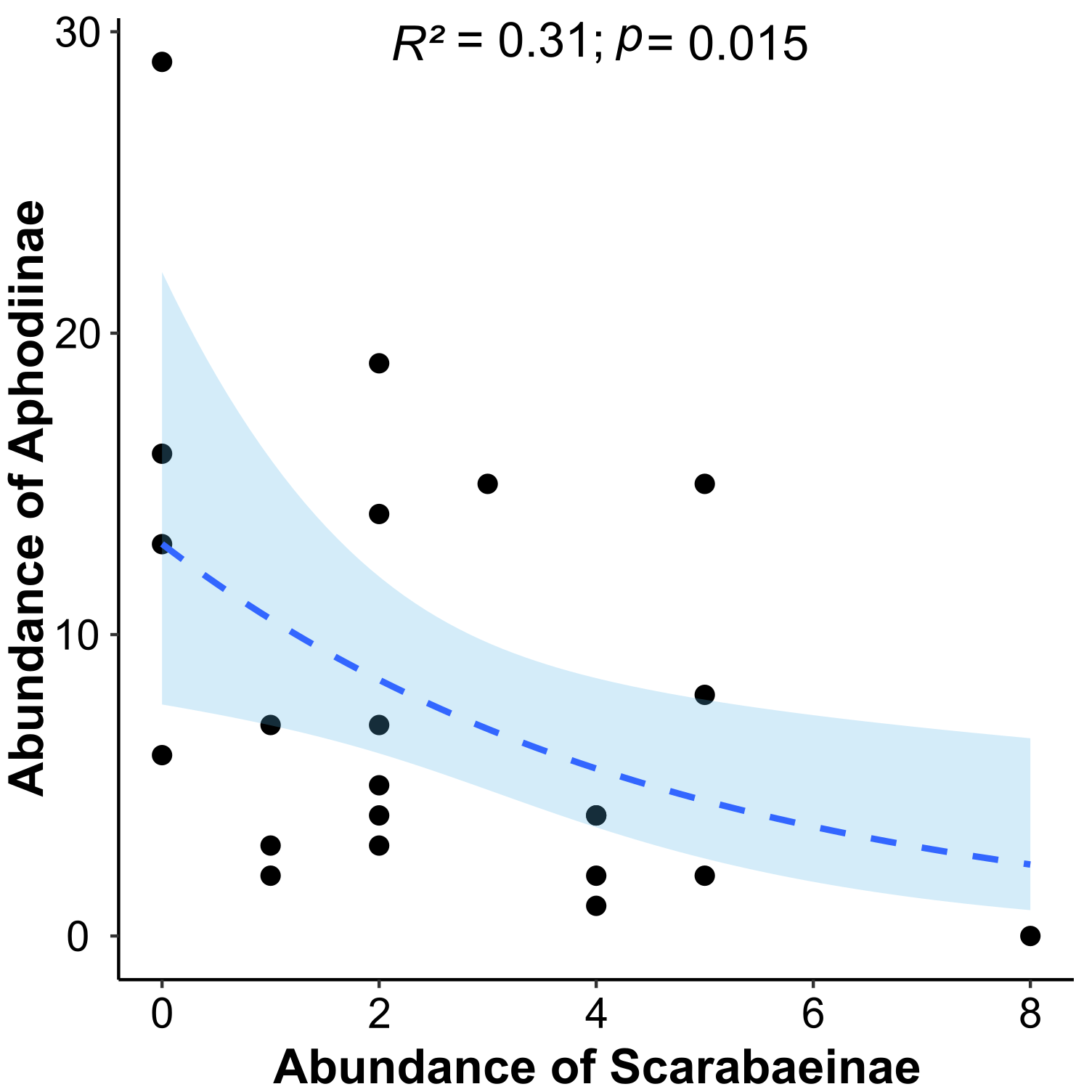


**Online Resource 10.** Relationship between the abundance of individuals in the Aphodiinae and Scarabaeinae subfamilies. We fitted a generalized linear model using a negative binomial error distribution due to overdispersion.
